# Microrheology for biomaterial design

**DOI:** 10.1063/5.0013707

**Published:** 2020-12-29

**Authors:** Katherine Joyner, Sydney Yang, Gregg A. Duncan

**Affiliations:** 1 Fischell Department of Bioengineering, University of Maryland, College Park, Maryland 20742, USA; 2 Biophysics Program, University of Maryland, College Park, Maryland 20742, USA

## Abstract

Microrheology analyzes the microscopic behavior of complex materials by measuring the diffusion and transport of embedded particle probes. This experimental method can provide valuable insight into the design of biomaterials with the ability to connect material properties and biological responses to polymer-scale dynamics and interactions. In this review, we discuss how microrheology can be harnessed as a characterization method complementary to standard techniques in biomaterial design. We begin by introducing the core principles and instruments used to perform microrheology. We then review previous studies that incorporate microrheology in their design process and highlight biomedical applications that have been supported by this approach. Overall, this review provides rationale and practical guidance for the utilization of microrheological analysis to engineer novel biomaterials.

## I. INTRODUCTION

Biomaterials are a versatile and diverse class of materials that have significantly advanced long-established and emerging fields such as drug delivery, tissue engineering, and immunoengineering.^1–3^ For these applications, precise control of their chemical and mechanical properties is critical to drive a specified biological response.^4^ Soft biomaterials are often used to mimic the viscoelastic properties of living systems.^5^ The ability to control cell differentiation, cell transport, and drug release all arises through the engineering of these materials on the microscale. Understanding these complex network architectures will determine if the desired cellular phenotype and/or biological effect will be achieved. As a result, microscale properties, such as network structure and adhesive ligand arrangement, are key parameters in the design of biomaterials. Measurement tools with access to properties on this scale could inform optimal biomaterial fabrication strategies.

Rheology is the study of how materials deform in response to force and is routinely examined in biomaterial systems.^6,7^ Traditionally, mechanical measurements of biomaterial constructs are carried out at the macroscale using a rheometer or a dynamic mechanical analyzer (DMA) and far less often using microrheological approaches. Microrheology can be used to investigate the internal microenvironment of soft materials, by observing thermally or field-driven movement of colloidal probes entrenched within the material.^8^ The motion of these particles can exhibit a linear or nonlinear response depending on the surrounding medium. This response can be quantified to understand material properties and mechanics.^9^ Microrheology has been used to characterize a wide range of complex fluids, suspensions, and soft polymeric materials.^10^ Using microrheology in the design of biomaterials has many practical advantages such as rapid acquisition speed, simple preparation requirements, and low sample volume.^11^ The high spatiotemporal resolution of microrheological measurements allows for micro- to nanoscale interactions and dynamics to be directly probed providing a significant technical advantage in biomaterial design.

In this review, we aim to provide the rationale for microrheology to be considered alongside standard techniques for biomaterial design. We first introduce the theory behind microrheology and the different methodologies available to biomaterial scientists. We next highlight how microrheology has been used to engineer unique features into biomaterials and as a high throughput screening tool. We also share previous works using microrheology to build novel materials with biological and translational relevance. Finally, we discuss the current trends in microrheology and prospects of further incorporating this technique into biomaterial design.

## II. OVERVIEW: MICRORHEOLOGY

### A. Background and theory

Rheological measurements are necessary to understand the mechanical properties of biomaterials resulting from the structures of their polymer networks.^12^ Biomaterials frequently exhibit viscoelastic behaviors, and using rheology, these properties can be examined as a function of time, force, and type of deformation.^5^ Using both macro- and microrheological techniques, previous work has clearly shown that the equilibrium and dynamic properties of biomaterials are the result of their microscopic and microstructural features (e.g., polymer chain mechanics, relaxation times, and dynamic assembly).^7,13–15^ As will be discussed throughout this review, understanding microscale viscoelastic properties can be an important aspect of biomaterial design as this will relate to many critical features such as heterogeneity of internal architecture, cell-scaffold interactions, and mechanisms of drug release. Microrheology enables researchers to understand these microscale mechanical properties by either monitoring (i) diffusion or (ii) external field driven transport of small colloidal probes embedded within the material. Using a variety of spectroscopy and microscopy-based techniques, microrheology has found broad utility in soft matter physics and colloidal science because of the unique perspectives and rich information able to be interrogated at short length and time scales.^16^

The fundamental basis for microrheology was established over two centuries ago, dating back to the 1800s, with experiments measuring the diffusion of suspended particles by Brown, Einstein, Perrin, and others. The motion of a particle within a fluid can be described using the Langevin equation as

$$m\left(\partial U/\partial t\right)={{\;F}_{B}+F}_{H}+{\;F}_{C},$$ (1)

where *m* is the particle mass, *U* is the particle velocity, *F*_B_ are stochastic Brownian forces, *F_H_* are hydrodynamic forces due to frictional drag, and *F*_C_ are conservative forces due to external fields (e.g., gravity, optical, or magnetic traps). Thus, at equilibrium, thermally driven, random Brownian movement of fluid suspended particles can be directly related to the forces (stress) exerted by the probe particles (*F*_B_) and the response of the surrounding environment (*F*_H_).^9^ Particle diffusion is most often quantified as a time-averaged (*t*) mean square displacement (MSD; $\left\langle \Delta r^{2}(t)\right\rangle$). For colloidal probes diffusing in 3D within viscous (Newtonian) liquids, probe particles will freely diffuse and the MSD will increase linearly with time,

$$\left\langle \Delta r^{2}(t)\right\rangle =6\mathrm{Dt}.$$ (2)

MSD will scale with the diffusion coefficient (*D*) of the probe, *D* = *k_B_T*/6πηa, where *k*_B_ is the Boltzmann constant, *T* is the temperature, *η* is the material viscosity, and *a* is the radius of the probe.^16^ We often note that microrheology measurements based on particle diffusion are performed in 2D using optical and fluorescence microscopy. If it is assumed that the material is locally isotropic, 2D measurements can be used for reasonable approximation of 3D diffusion coefficients. To establish a relationship for particle diffusion in a surrounding medium with time scale-dependent viscoelastic properties, Mason and Weitz introduced the generalized Stokes–Einstein relationship (GSER),^17^

$$\tilde{G}\left(s\right)=\frac{k_{B}T}{\pi \text{as}\left\langle \Delta r^{2}(s)\right\rangle },$$ (3)

where *s* is the Laplace frequency, $\tilde{G}\left(s\right)$ is the Laplace transformed relaxation modulus, and $\left\langle \Delta r^{2}(s)\right\rangle$ represents the Laplace transformed MSD. Frequency (*ω*)-dependent storage (*G′*) and loss modulus (*G″*) can be defined by substituting *s* with *iω* as shown in Eq. (3),^18^

$$G^{*}\left(\omega \right)=G\prime \left(\omega \right)+\mathrm{iG\prime \prime}.$$ (4)

As mentioned, microrheology is uniquely suited to measure local structural and dynamic properties of biomaterials. For example, the pore size (ξ), a critical parameter for molecular diffusion and cell migration in biomaterials, can be estimated based on the MSD [Eq. (5)] or *G′* [Eq. (6)],

$$\xi \;\approx \;\sqrt{\left(\left\langle \Delta r^{2}(t)\right\rangle \right)+a}$$ (5)

or

$$\xi \approx {\left(\frac{k_{B}T}{G^{\prime }}\right)}^{1/3}.$$ (6)

We have demonstrated that these two estimations provide similar pore size dimensions.^19^ Bulk rheometers are inherently disruptive during assembly and may potentially interfere with reversible network association in self-healing soft materials. In contrast, microrheology is capable of probing assembly dynamics throughout liquid–solid and solid–liquid transitions in soft materials. The gel point can be determined based on the power law exponent [α; Eq. (7)] of the mean-squared displacement over time (τ),

$$\alpha =\frac{\;{\text{log}}_{10}\;(\langle \Delta r^{2}(t)\rangle )}{\;{\text{log}}_{10}\;(t)}.$$ (7)

A value of α equal to 1 is indicative of a purely viscous environment, and for purely elastic substances, the value of α approaches 0. For a gel network, subdiffusive probe movement should be observed within the viscoelastic environment, where 0 < α < 1. A threshold exponent, *n*, can be defined to determine whether the material is a gel, where α < *n,* or a liquid, where α > *n*. The value of *n* can be determined using time-cure superposition analysis of acquired microrheology data where this value will vary in magnitude dependent on network connectivity.^8,20–22^

While practically simple and the most widely used approach, relying on the random, thermally driven movement of probes alone to characterize biomaterial microrheology generally limits analysis to linear and equilibrium behaviors. To extend the parameter space that may be interrogated, active microrheology can be employed where particle probes are driven through a material of interest by the application of a constant or oscillatory external force several orders of magnitude stronger than thermal fluctuations (*F*_C_ ≫ *F*_B_). In this scenario, the probe particle may no longer be in equilibrium where the magnitude and direction of *F*_C_ are often a function of time. Traditionally, active microrheology experimental setups consist of an optical or magnetic trap to direct probe particle motion and an optical or fluorescence microscope for tracking of probe motion. In a manner analogous to bulk rheology measurements, active microrheology can be used to study nonlinear viscoelastic responses of a material by measuring phase differences in deformation following oscillatory applications of force.^8^ Shifts in the phase angle (Δϕ) between the maximum measured force (*F*_max_) and displacement of the trapped probe (*x*_max_) over a range of ω values can be used to determine the storage and loss moduli as shown in the following equations:^23^

$$G\prime \;\left(\omega \right)=[F_{\text{max}}/6\pi {\mathrm{Rx}}_{\text{max}}]\;\;\text{cos}(\Delta \phi ),$$ (8)

$$G\prime \prime \left(\omega \right)=[F_{\text{max}}/6\pi {\mathrm{Rx}}_{\text{max}}]\;\;\text{sin}(\Delta \phi ).$$ (9)

### B. Methodology

A number of techniques exist to perform colloidal probe-based microrheology including dynamic light scattering (DLS), diffusing wave spectroscopy (DWS), multiple/single particle tracking (MPT/SPT), and optical/magnetic tweezers (OT/MT). Light scattering techniques (e.g., DLS and DWS) provide an ensemble average of microscale properties, whereas microscopy methods can provide single-particle level information to enable local characterization of biomaterial viscoelasticity. These methods can be subdivided into passive approaches, based solely on probe diffusion, and active approaches, where external fields are used to direct and control probe motion. Specific examples with their benefits and limitations are highlighted in Table I. In this section, we will provide a brief overview of each approach and recommend the references cited in Table I for further details.

**TABLE I.** Summary of the benefits, limitations, and operating regime of colloidal probe-based microrheology techniques such as dynamic light scattering (DLS), diffusing wave spectroscopy (DWS), multiple/single particle tracking (MPT/SPT), and optical/magnetic tweezers (OT/MT).

Technique (passive/active)	Benefits	Limitations	Frequency range (rad/s)	Max elastic moduli (Pa)	References
DLS (passive)	Broad frequency range Common equipment	Limited to transparent samples	10^–1^–10^5^	10^3^	24 and 27
DWS (passive)	Widest frequency range utilized for opaque samples suitable for stiffer materials	Large volume requirements, specialized equipment needed, and sensitive to probe the particle concentration	10^0^–10^7^	10^5^	17 and 26
MPT and SPT (passive)	High (typically) 2D spatial resolution. Simple setup.	Limited to soft materials at equilibrium, not ideal in active systems.	10^0^–10^3^	10^2^	18 and 30
OT and MT (active)	High, 3D spatial resolution, ideal for shear-thinning (nonlinear) materials	Specialized equipment needed	10^0^–10^5^	10^4^	5 and 32

**FIG. 1.**
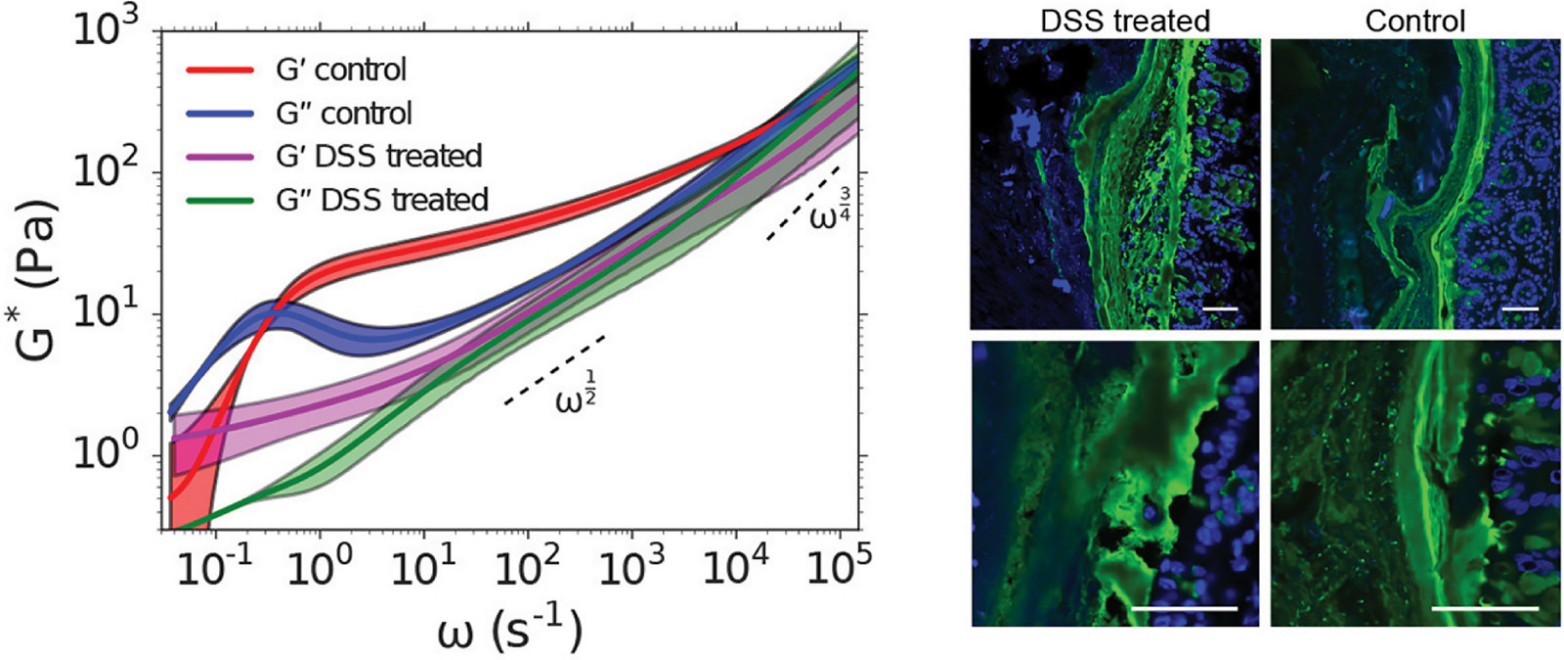
Dynamic light scattering (DLS) microrheology. (Left panel) Frequency-dependent mechanics of intestinal mucus from healthy and dextran sulfate sodium (DSS)-induced colitis mice as determined using DLS microrheology. (Right panel) Confocal imaging of mucus (green) in the colon of DSS-treated and healthy mice. Reproduced with permission from Krajina *et al.*, ACS Cent. Sci. **3**, 1294 (2017). Copyright 2017 American Chemical Society. Further permissions related to the material excerpted should be directed to the ACS.

Dynamic light scattering (DLS) relates the fluctuations in probe scattering intensity to particle displacement or MSD to material properties. The MSD is derived through an autocorrelation function of measured scattering intensities from which viscoelastic moduli can be determined using GSER [Eqs. (3) and (4)].^17^ A benefit of DLS is the ability to probe a wider range of frequencies compared to traditional rheological methods, which typically range from 10^0^ to 10^2^ rad/s. For example, DLS microrheology has enabled determination of the elastic and viscous moduli of polyacrylamide gels in frequencies ranging from 10^−1^ to 10^3^ rad/s using DLS.^24^ Krajina *et al.* have developed a user-friendly DLS microrheology workflow that utilizes backscattering to capture even broader time scales ranging from 10^−1^ to 10^6^ (*ω*) s^−1^ at higher probe concentrations and lower sample volumes than previously realized using DLS methods.^25^ To demonstrate the utility of this approach, healthy and diseased mouse intestinal mucus was characterized *ex vivo* and in diseased animals, measured changes in mucus viscoelasticity corresponded to the loss of mucosal barrier function (Fig. 1).

Diffusing wave spectroscopy (DWS) is another light scattering methodology sensitive to the passive motion of probe particles. In contrast to DLS where the phase differences of single particle scattering are observed, DWS accounts for the collective effects of multiple particle scattering in the sample. This approach extends light scatting microrheology to turbid or high scattering samples.^26^ Multiple scattering events are a function of probe motion on short length (on the order of nanometers) and time scales, making DWS suitable for high frequency measurements.^27^ Similar to DLS, MSD can be determined through an autocorrelation function of scattering intensities and GSER can be applied to determine frequency-dependent viscoelastic properties.^28^ Using this method, viscoelastic properties of cytoskeletal filament networks and the effects of surfactant additives were determined using DWS and other rheological methods. The use of DWS significantly increased the range of frequencies measured above 10^5^ rad/s, highlighting the frequency-dependent response of the material.^29^

**FIG. 2.**
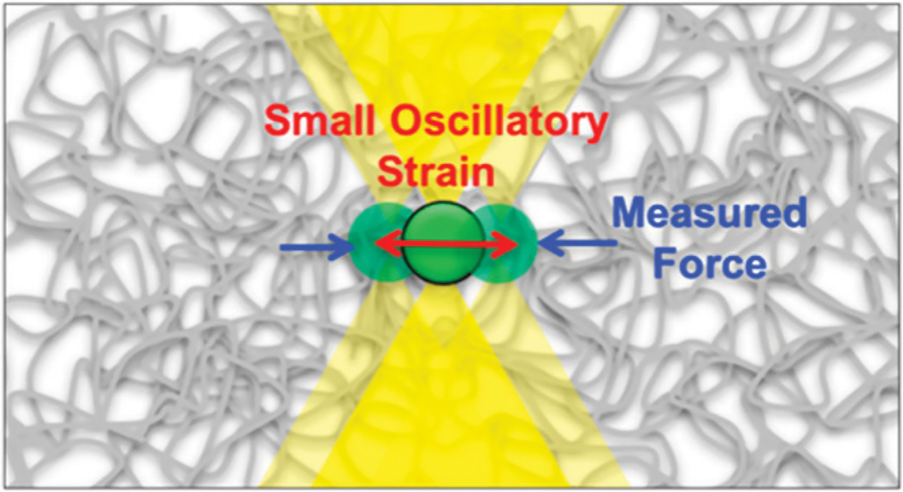
Optical tweezers for active microrheology. Optically trapped probes used to exert oscillatory (as pictured) or constant strain on material of interest. Measured force and particle displacement are used to determine the frequency-dependent mechanical response. Reproduced with permission from R. M. Robertson-Anderson, ACS Macro Lett. **7**, 968 (2018). Copyright 2018 American Chemical Society. Further permissions related to the material excerpted should be directed to the ACS.

Microscopy methods are broadly used for microrheology, because of the relatively simple setup with standard microscopes and readily available tracking software packages. These methods of microrheology are typically divided into passive or active approaches, which measure thermally or externally driven probe motion, respectively. Passive approaches include multiple (MPT) and single particle tracking (SPT), which require a simple setup for data acquisition.^30,31^ However, given the movement of probe particles is driven by *kT*-scale thermal fluctuations, this approach is typically limited to soft materials. Active microrheology can extend the range of accessible elasticities by applying an external force to the probe. An example of OT-based microrheology is illustrated in Fig. 2 where the viscoelastic properties can be extracted from the differences in the small oscillatory strain of the measured force and probe displacement. Given its ability to probe nonlinear material responses, active microrheology can be very useful to understand yield stress and shear-thinning properties of biomaterials.^9,23,32^ In addition, active microrheology can be exploited in analyzing active biological systems (e.g., cell-laden hydrogel scaffolds). It is important to note, however, that many biomaterials with greater stiffness requirements for applications such as bone tissue engineering and surgical adhesives, are beyond the range of elastic moduli (>100 kPa) that can be assessed using passive or active microrheology.^33^ Atomic force microscopy (AFM) provides a useful alternative in this scenario where similar analyses of dynamic and spatially dependent biophysical properties can be assessed.

### C. For beginners: General considerations for particle tracking microrheology

As the most widely used and most accessible to nonexperts, we provide here a guide for those beginning to incorporate conventional particle tracking microrheology into their research and common pitfalls in this approach that can be avoided with appropriate experimental design. For passive microrheology, the motion of embedded colloidal probes in a material is sensitive to its interactions with the environment. The probe size and surface chemistry will significantly influence these interactions and, consequently, the experimental outcomes. Therefore, these features of the probe should be a primary consideration for any microrheology experiment. Probe sizes typically range from 0.1 to 10 *μ*m microspheres. For ease in interpretation, probes should also be uniform in size, spherical in shape, chemically stable, well-dispersed throughout the material, and nondisruptive to the sample.^8^ When measuring the viscoelastic properties of biomaterials, the probe size is an important consideration as it will depend on the elasticity and polymer mesh spacing within the biomaterial.^28,34^ As a function of length and time scale, cross-linking and entanglements within the polymer network will influence the probe's displacement.^35^ For example, when the probe radius, *a*, is larger the network mesh size, *ξ* (or *a* ≫ *ξ*), its diffusion will follow a linear time-dependent viscoelastic response as specified by the GSER. However, if *a* < *ξ*, the probe will diffuse through the mesh and depends strongly on the network architecture. At high enough elasticities (e.g., >10^2^ Pa for 0.1 *μ*m probes), the probe will become immobilized and unable to sample its environment. Thus, this form of microrheology is most appropriately used for soft biomaterials such as hydrogels.

The composition and surface chemistry of the probe are also important experimental considerations. Probes are commonly fabricated using materials such as polystyrene, silica, or iron. These are commercially available and may be loaded with fluorescent dyes that provide an excellent signal-to-noise ratio ideal for optical imaging. Probe surface chemistry can significantly alter the diffusion of probes within biomaterials.^36,37^ Probes that are adherent to the gel network often experience viscoelasticity on the order of what is measured using bulk rheological methods.^38^ Conversely, probes that are modified to render their surfaces nonadherent to biomaterials experience microviscosity several orders of magnitude lower than their adhesive counterparts, are sensitive to changes in the network microstructure, and may detect alterations in the viscosity of the interstitial fluid phase.^36^ Changes in surface chemistry can be made via covalently attached or adsorbed coatings (e.g., Pluronic, bovine serum albumin). Covalent attachment of dense coating of poly(ethylene glycol) (PEG) is a commonly used particle surface modification.^39^ PEG surface coatings render the probes' surface hydrophilic, neutrally charged, and generally nonadhesive to biological polymers. Probes that undergo aggregation within the biomaterial will compromise accurate microrheological analysis and should be avoided.^36^ It is also critical to properly seal microscopy samples to prevent drying or unwanted gas exchange.

Tracking the embedded probes with the spatial and temporal resolution required for microrheology requires image acquisition at high magnification (e.g., ≥63×) using cameras equipped for high acquisition speed (e.g., millisecond exposure times).^40^ Subpixel spatial resolution, on the order of nanometers, can be achieved using an image analysis algorithm developed by Crocker and Grier.^41^ However, it is important to account for error even in quality images with high signal-to-noise ratios. Two major types of errors include static error and dynamic error.^42^ Static error is attributed to the intrinsic variation in the experimental setup such as changes in fluorescence intensity or vibrations of the microscope stage. Tracking data can be corrected for static errors by measuring the MSD of completely immobilized particles used in the experiment. Kowalczyk and colleagues demonstrated this method and noted that the degree of error was dependent on the particle size.^43^ Dynamic error is a result of the mismatches in image acquisition speed and the characteristic time scale of particle motion. Adjusting for dynamic error is achieved by comparing MSD with a model of expected MSDs;^44^ however, it is often difficult to accomplish this in polymeric materials with heterogeneous properties. Dynamic error can be minimized by considering data at mid-range to longer time scales, typically on the order of seconds.^45,46^ By performing these calibration steps, optimal experimental settings can be determined that effectively eliminate both static and dynamic error.

## III. MICRORHEOLOGICAL APPROACHES TO BIOMATERIAL DESIGN

### A. High-throughput screening for biomaterial formulation

Rapid high-throughput screening approaches can be used to design biomaterials for their use in numerous biomedical applications.^47–53^ For example, extracellular matrix (ECM) microarrays have been fabricated with 741 combinations using 38 different signaling factors to examine endoderm cell differentiation into hepatic and pancreatic cells.^54^ Using multiwell arrays, the functionality of hydrogel scaffolds with varying stiffness and density values of cell adhesion ligands was examined based on human mesenchymal stem cell (hMSC) proliferation.^55^ These types of high-throughput screening approaches, using biological activity as a readout, streamline the biomaterial formulation process but do not provide direct information on the physicochemical properties of the biomaterial itself. Traditionally to capture this, bioanalytical techniques, such as mass spectrometry and chromatography, can be coupled with bulk rheological testing to characterize biomaterial formulations.^12,56^ However, bulk rheological approaches are difficult to perform in a high-throughput manner and provide limited information about the local microenvironment within the biomaterial. Microrheology is uniquely suited to probe these important features in a high-throughput manner and has the practical advantages of acquisition speed, simple sample preparation, and minimal volume required for the characterization of large biomaterial libraries.^11^

Several groups have demonstrated the use of microrheology for screening of biomaterial designs. For example, microrheology experiments were combined with a microfluidic device to achieve a fast and efficient method of forming high-molecular weight heparin (HMWH)-PEG hydrogels while characterizing their viscosities as a function of polymer concentration.^57^ Spero *et al.* have also engineered a magnetic high throughput screening microplate that is scalable to a 96-well format.^58^ The embedded tracer particles necessary for microrheology also have the sensitivity to capture microscale phase transitions of the network during assembly and disassembly. For example, Escobar *et al.* were able to measure gel–sol transitions [using Eq. (7)] of covalent adaptable hydrogel scaffolds to reveal degradation mechanisms of at physiological pH.^59^ Using microrheology and measured α as a marker, mix-induced gelation of novel peptide biomaterials was examined as a function of concentration and ratio of protein domains.^60^ Combining microrheology for measurements of biomaterial properties by high-throughput 3D cell-culture screening technologies could also provide a powerful screening tool with readouts on both cell and biomaterial functions. For example, a high-throughput screening tool was developed for malignant pediatric brain drug screening in 3D *in vitro* cancer models using a peptide-based hydrogel as a 3D scaffold.^61^ Incorporating microrheology into this screening could reveal other mechanical changes to these scaffolds during drug treatment, which could provide information relevant to tumor pathology.^62^

Automation is a key feature in any high-throughput screening tool. Specifically, for particle tracking microrheology, there are several methods to automatically perform particle selection and tracking.^63–65^ However, these methods still require user input to set pixel intensity thresholds, validate particle selection, and adjust for errors. Each instance of user input increases the data processing time of each experiment and, importantly, may introduce user-associated errors. To overcome these limitations, several advances have been made toward providing increased accuracy and full automation in the analysis of particle tracking data. Curtis *et al.* have introduced the open source parallel computing approach, diff_classifier, which can increase speed and accuracy of data analysis.^66^ This is achieved by using cloud computing technology and expanding the tracking parameters beyond intensity-based particle detection. To further increase automation in particle identification, deep learning computational techniques are being introduced. For example, a neural network algorithm was trained using simulated 2D and 3D particle tracking data that substantially decreases processing time and required no user-defined parameters.^67^ Traditional tracking analysis can bias toward longer trajectories, limiting accuracy and increasing the number of consecutive frames needed. Another deep learning algorithm was developed to acquire diffusion data from short particle trajectories, which again will increase accuracy and decrease the acquisition time.^68^ These technical advancements in particle tracking automation could further improve the use of microrheology in high-throughput systems.^69^

### B. Network heterogeneity and engineering mechanical gradients

Many biological hydrogels naturally possess heterogenous properties due to variations in network assembly, which are important for their function. For example, in the extracellular matrix (ECM), heterogeneity in the scaffold microstructure can strongly influence interactions with living cells in processes such as cell-ECM adhesion, cell migration, and ECM remodeling.^70–72^ As such, it is often desirable to produce biomaterials with anisotropic mechanical properties in order to mimic *in vitro* the microenvironment in native tissues *in vivo*.^33,73,74^ Traditionally, this is achieved using light-based approaches allowing stepwise controlled photopolymerization and spatial variation in the degree of cross-linking.^75,76^ For example, PEG and hyaluronic acid (HA) 3D cell culture scaffolds functionalized with diacrylate groups have been fabricated to enable UV-induced cross-linking in the presence of a photoinitiator.^77^ Light-based cross-linking of hydrogels can enable spatially controlled stiffness down to the micrometer scale.^78^ In order to characterize these biomaterials, techniques capable of capturing the spatial dependence in rheology are required to ensure successful establishment of the desired physical properties.

Standard rheological methods only provide information on the average mechanical properties and cannot provide direct information on structural heterogeneity. In addition, the degree of swelling and swelling ratios only offer a bulk assessment of the hydrogel network size. In order to quantitatively characterize spatially dependent changes to viscoelasticity, techniques such as atomic force microscopy (AFM) have been used to map changes in Young's modulus.^79,80^ AFM can be used to directly quantify both network stiffness as a function of spatial position and the spacing between individual polymer fibers.^81–83^ Unlike AFM, which is traditionally limited to topographical measurements at the biomaterial surface, active microrheology is also advantageous as it enables extraction of mechanical properties using probes embedded within biomaterials with precise control of their spatial position. Another important feature of both AFM and active microrheology using OT/MT is that they do not require drying or snap-freezing for assessment. This is a significant limitation of using scanning electron microscopy (SEM) or cryo-SEM, respectively, which may alter the natural structure of biomaterials due to this processing.^75^

**FIG. 3.**
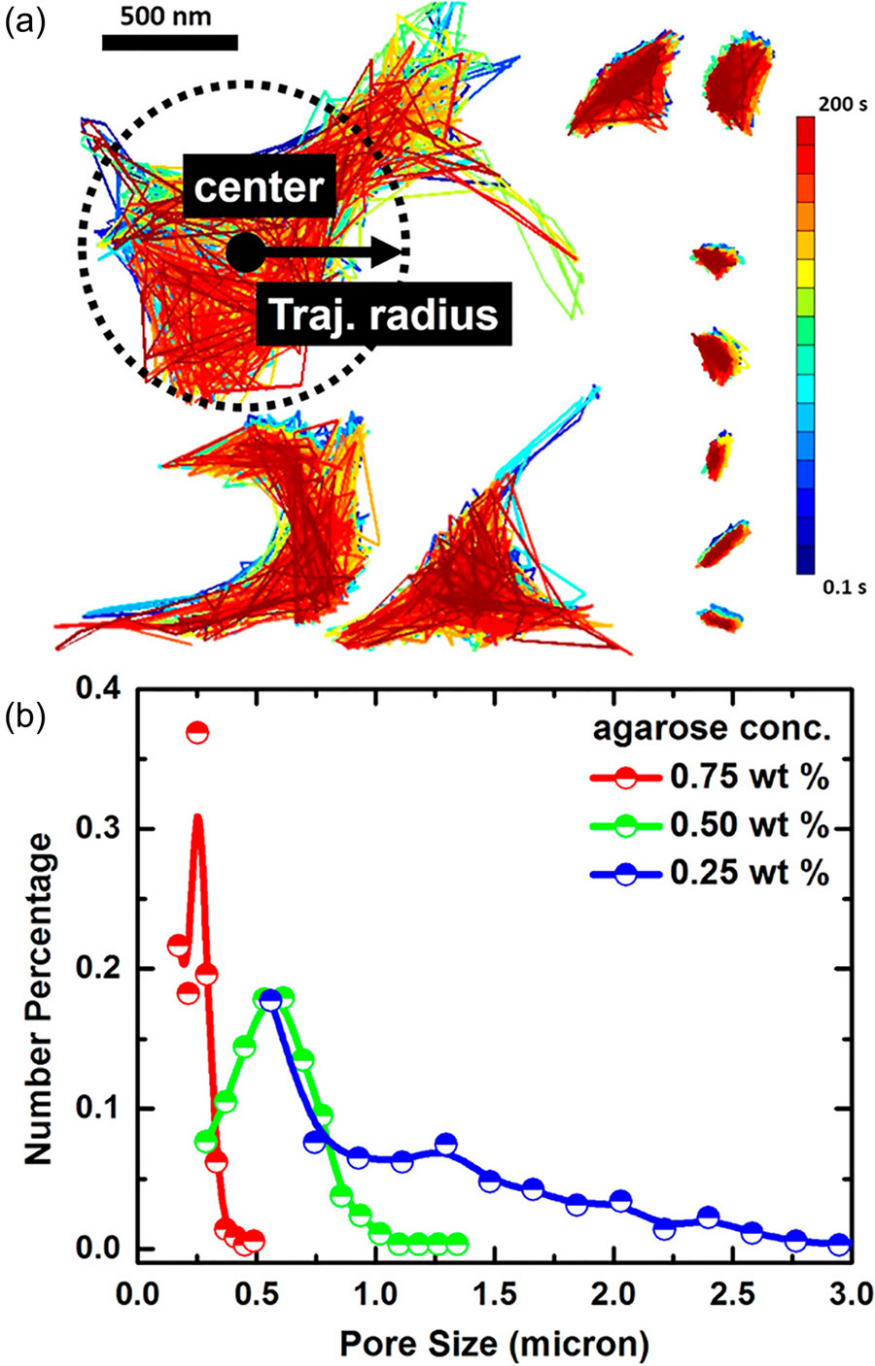
Visualization and direct measurement of biomaterial network geometry. (a) Porosity of agarose gels was characterized based on individual trajectories determined using particle tracking microrheology. (b) Based on this analysis, pore size distribution were determined as a function of agarose concentration. Reprinted with permission from L. Jiang and S. Granick, ACS Nano **11**, 204 (2017). Copyright 2017 American Chemical Society. Further permissions related to the material excerpted should be directed to the ACS.

It has been demonstrated in previous work that the heterogeneity in hydrogel properties can be determined using microrheology. Microrheological measurements have been performed in biomaterials such as agarose, gelatin, and F-actin where particle mobility was non-Gaussian as a result.^34^ The non-Gaussian behavior observed can be attributed to local differences in microviscosity and spacing between biopolymer fibers. Visualization of individual probe particle trajectories within these matrices revealed the spatial heterogeneity in microrheological properties. Further validating this approach, Shin *et al.* provided direct evidence that microrheology provided comparable network size measurements to electron microscopy-based analysis of the F-actin network structure.^84^ As a result of this work, a simplified physical model was developed to directly translate measured MSD to an effective pore network size [Eq. (5)]. More recent efforts have developed the means to spatially visualize and measure the pore network structure based on the diffusion of nano- and microparticles within porous scaffolds (Fig. 3).^85,86^ Typically, particle tracking measurements are conducted over short times, on the order of seconds, and the collective movement of colloidal probes is averaged to reduce the inherent noise of individual particle diffusion prior to analysis. To spatially map the pore structure based on individual trajectories, nano- and microparticles within porous media must be tracked over long times, on the order of minutes, in order to generate sufficient statistics to reliably assess spatial variation in network dimensions.^85,86^

Biomaterials commonly used for 3D cell culture such as collagen and matrigel have previously been studied using microrheology. Using optical tweezers microrheology, the concentration and temperature dependence of collagen gel microviscosity and the network structure were assessed.^87,88^ Using this approach, they found the 10- to 25-fold differences in elastic and shear moduli, respectively, at different positions within collagen gels, indicative of the microscale heterogeneity of gel properties. In recent work, heterogeneity in the microscale architecture of collagen gels, visualized through reflectance imaging, was further confirmed to contribute to local changes in gel stiffness.^89^ Specifically, the elastic moduli locally increased in regions of high collagen fiber density. The impact of probe particle surface chemistry and solvent conditions in matrigel and collagen were explored showing that probe-matrix interactions were dependent on both the network size and the ionic species, which strongly influenced particle diffusion.^90,91^ It should be noted that the use of particle probes with matrix adhesive surfaces (e.g., negatively charged particles in the case of net-positively charged matrigel at physiological pH) will limit their ability to probe the mesh size of the gel.

Unlike in AFM, microrheology can also be adapted to determine the 3D spatial changes in mechanical properties. As noted previously (Sec. II B), particle tracking microrheology in hydrogels is typically performed in 2D and measured properties are extrapolated to 3D. However, performing microrheology measurements in 3D may be desirable depending on the nature of the engineered mechanical gradients. Moving from 2D to 3D microrheology presents some technical challenges, which may be addressed with a few modifications. For passive microrheology, 2D particle tracking can be paired with a motorized stage with control of the depth or z-position within the gel. To directly measure 3D trajectories of probe particles, advanced imaging techniques, such as orbital tracking and holographic video microscopy, have been employed.^92–94^ Using optical or magnetic tweezers-based approaches, the probe particle position can be directly controlled and, thus, enables spatial mapping of biophysical properties. As an additional feature unique to the microrheological approach, intracellular microrheology may be performed *in situ* to determine if biophysical changes to the cell occur in response to gradients in matrix stiffness.^95^ In Secs. IV A and IV B, we will discuss recent examples of microrheology within cell-laden biomaterial scaffolds.

### C. Engineering stimuli-responsive biomaterials

Biomaterials can be designed to respond to stimuli such as temperature, pH, solvent conditions, and enzymatic species where these stimuli can either promote formation or degradation of the material. Many gel-forming biomaterials will naturally or are engineered to undergo sol–gel transitions in response to pH (e.g., neutral to acidic pH) or temperature (e.g., room to body temperature). For example, gelation of naturally occurring biomaterials such as chitosan and alginate, both carrying net charge, may occur in response to pH conditions and specific ionic species.^96,97^ Collagen undergoes a sol–gel transition at 37 °C as fibers rapidly assemble into a gel network.^72^ In addition, collagen gels and other tissue culture scaffolds may be degraded by enzymes secreted by cells embedded within the matrix.^71^ Dependent on the application, understanding whether these stimuli enable reversible assembly and disassembly may also be of interest.

Designing rheological measurements to capture these changes can be challenging using standard techniques depending on the time scale of interest and source of the stimuli. For example, bulk rheological measurements may be carried out to characterize pH- and/or temperature-responsive biomaterials, but it is challenging to probe the responses on the short time scales relevant to biomedical applications. For an injectable temperature-sensitive biomaterial formulation, it may be desired for the material to rapidly form, within seconds to minutes, once it comes into contact with the target tissue.^98,99^ Small differences in the time to gel will then make a large impact on the overall performance, and thus, standard techniques may not provide the information necessary to determine an optimal formulation. It would be desirable to rapidly perform these measurements in order to capture these transitions in real time. To examine enzymatically degradable biomaterials using a traditional rheometer, proteases may be added exogenously to recapitulate cell-mediated degradation. However, with the protease added homogeneously to the gel, the dynamics and/or spatial distribution of biomaterial degradation will likely differ from that of proteases secreted locally by cells within the matrix, which has been shown to influence cell behavior.^77,100,101^

Toward this end, microrheology can offer the means to address these shortcomings as has been demonstrated in several relevant biomaterial systems. For example, the temperature-dependent sol–gel transition of collagen has been examined in previous work where gels of varying collagen concentrations showed notable changes in fiber morphology and network heterogeneity.^87,88^ Jiang and Granick demonstrated how gel formation and network structure heterogeneity in agarose gels respond to changes in temperature using microrheology.^86^ Interestingly, they also demonstrated that they could study reversibility of gel formation upon repeated heating and cooling cycles. This has been similarly examined for DNA hydrogels that are responsive to temperature using particle tracking, DLS, and DWS microrheology.^102,103^ Formation of UV-activated acrylate hydrogels has also been observed, enabling the precise measurement of gelation times on the order of seconds as a function of polymer concentration and UV intensity.^104^ Upon addition of a proteolytic enzyme, degradation of peptide-functionalized PEG scaffolds has been monitored *in situ* using microrheology, where the resulting changes in network architecture and the uniformity of degradation could be directly assessed.^105^ Conversely, the gelation time of an enzyme-catalyzed peptide scaffold was observed using microrheology, forming in seconds.^106^ As a result of gradual reductions in pH over the course of hours triggered by addition of glucono-δ-lactone, fluorenylmethoxycarbonyl-tyrosine (Fmoc-Y) peptides assembled into gels with highly consistent network sizes as observed by microrheology.^107^

**FIG. 4.**
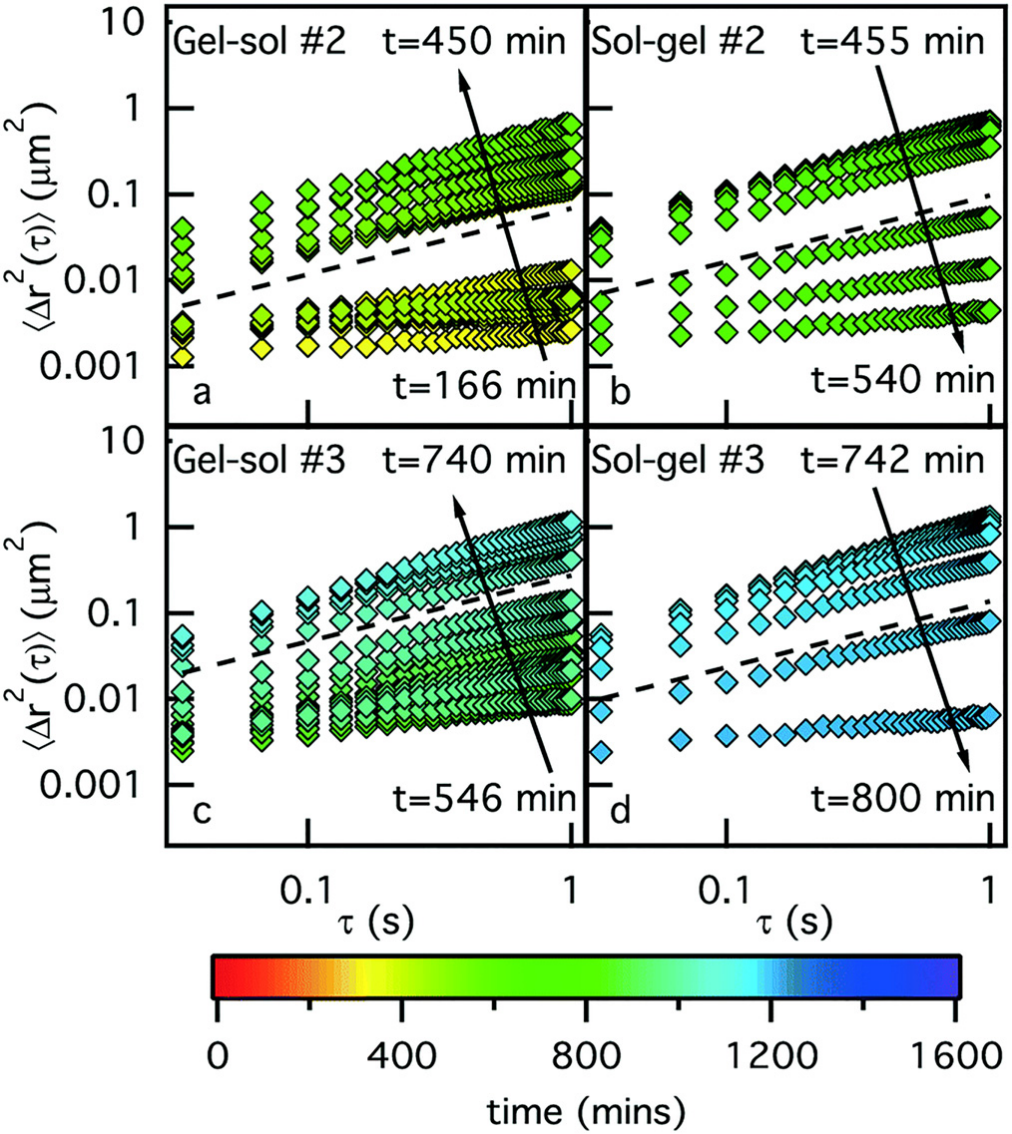
Microrheological analysis of sol–gel transitions. A microfluidic device coupled with particle tracking microrheology used to monitor reversible, osmotic pressure-induced gelation and degradation of fibrous colloidal gels composed of hydrogenated castor oil. MSD of embedded probes was periodically measured during solvent exchange with the gelling agent (glycerin) and water where the color bar indicates the current time of experiment. Republished with permission from Wehrman *et al.*, Lab Chip **17**, 2085 (2017). Copyright 2017 Royal Society of Chemistry, Clearance Center, Inc.

Advanced experimental setups have also been developed to enable studies where solvent conditions can be dynamically changed and rheological properties can be simultaneously monitored using microrheology. Using a novel microfluidic device capable of dialyzing hydrogels and being imaged for particle tracking, Sato and Breedveld demonstrated how alginate gels undergo sol–gel transitions in response to Na^+^ and Ca^2+^ on the order of minutes.^108^ Given the long equilibrations times of gels with varying ionic compositions required for bulk rheology, these rapid changes could, otherwise, not be resolved. More recent work by Wehrman *et al.* (Fig. 4) has also used a microfluidics-based approach to monitor microrheology in colloidal hydrogels in response to cycling changes in osmotic pressure by varying solvent conditions.^109^ We also propose that microrheology could be paired with pH and/or specific ion (e.g., Ca^2+^ and Mg^2+^)-sensitive dyes in order to spatially map changes in the solvent composition and local viscoelasticity.

## IV. NOVEL BIOMATERIALS DESIGNED THROUGH MICRORHEOLOGY

### A. Physiological models

Biomaterials are engineered to provide a cellular microenvironment that more closely mimics physiological conditions. This biomimicry is achieved by tuning the physicochemical properties of the scaffold to resemble the tissue of interest.^74,110^ Biomaterials that recapitulate the functions of ECM can be used as disease models, for drug screening, or as model tissues to support cell growth and differentiation toward a specific phenotype. In addition, engineered biomaterials provide a controlled and reproducible system for *in vitro* studies. For example, PEG hydrogels enriched with ECM components were designed as models of intestinal crypts and were successfully able to support intestinal stem cell proliferation.^111^ Models of the tumor immune microenvironment have also been developed to better understand cancer-immune interactions, in order to enhance immunotherapy efficacy.^112^ The biophysical properties of these complex models have been effectively characterized using microrheology.

Biomaterials have been used extensively to model the complex chemical and mechanical tumor microenvironment (TME) also known as the tumor niche.^113^ Using microrheology to probe TME models has provided the field with unique insight into the mechanics of tumor cell progression. For example, optical trap-based active microrheology was used to measure the local mechanical properties of mammary cancer cells seeded in a hyaluronic acid (HA) hydrogel. By interrogating the local and distant differences in stiffness of the microenvironment, the mechanical adaptive nature of malignant and nonmalignant cells was revealed.^114^ Using collagen as a matrix, Bloom *et al.* developed a multiple-particle tracking assay to map ECM deformation by fibrosarcoma cells.^115^ Ashworth *et al.* also used microrheology to measure changes in viscosity during the fabrication of customizable disease-relevant peptide hydrogel tissue scaffolds.^116^ Through the decoupling of mechanical and chemical cues during design, these materials were used to model the influence of the microenvironment on breast cancer cells. It was also noted that microrheological measurements were not influenced by the initial force applied from the rheometer as seen in bulk measurements, highlighting the benefit using microrheology in design of model tissues.

Mak *et al.* developed a unique mitochondria-tracking-based microrheology approach to assess the local fluctuations and spatial biomechanics in a 3D TME model.^117^ Through this study, the impact of 3D culture on intracellular microrheology was examined, providing additional insight into the dynamic modulation of cancer cell mechanics. They demonstrated using tracer mitochondria that “particles” enabled an even spatial distribution throughout each cell and provided comparable sensitivity in detecting local fluctuations compared to traditional particle tracking microrheology.^110^ Thus, microrheology is not limited to the use of synthetic particles and naturally derived tracer particles may also be utilized effectively as probes. Cancer organoid models with dynamic, anisotropic mechanical properties have also been studied using microrheology. Han *et al.* utilized microrheology to characterize the mechanical properties of a developing cancer organoid. The stiffness and heterogeneity of the cell population increased as the tumor progressed.^118^ Microrheology was shown to be capable of distinguishing distinct dynamic behaviors for specific cell subpopulations within the organoid. By characterizing the distinct mechanical behavior of single cells, this information can be used to understand the heterogeneity in phenotypic responses that are of critical importance to understand cancer metastasis. Hence, to understand TMEs, microrheology can be a useful tool used to gain insight into the mechanical properties of the local environment.

Mucus is a natural extracellular barrier that protects the body by preventing the transport of undesirable particles to the underlying cell surfaces.^65,119^ Models of this natural biomaterial are critical for various applications in biology and medicine. To study its functional properties, mucus can be harvested from tissues, collected from culture models, or prepared from purified mucin-based gels. However, these approaches require difficult extraction procedures and costly production and contain significant variability between batches.^120^ Recently, our group used microrheology to design and characterize a physiologically relevant mucus model.^19^ Using multiple particle tracking microrheology, formulations of porcine gastric mucin and varying geometries of PEG-thiol cross-linkers were screened based on nanoparticle MSD. The microstructure of mucus is an important barrier property of the material and is well-known based on measurements in native, *ex vivo* mucus. Unlike traditional bulk assessments, direct comparison of biomaterial models and human samples, available in limited quantities, can be uniquely captured using microrheology performed on small volumes of patient-derived material. In our own previous work, we compared measured microstructural properties with these previous studies of human mucus and, using Eqs. (4) and (5), confirmed that our mucin-based hydrogels possessed similar network sizes.^19^

### B. Biomedical applications

Given their versatile properties, biomaterials are also being used in applications such as tissue engineering, wound healing, and drug delivery. For regenerative medicine applications, bioengineered tissue scaffolds that promote cellularization and organization into native-like tissues are widely sought after. As such, hydrogel scaffolds with properties similar to native ECM provide a realistic means to create a suitable niche for tissue regeneration. In addition to incorporating native bioactive species (e.g., cell adhesion ligands and growth factors), it has been shown that cell differentiation can be strongly influenced through 3D physical cues provided by the scaffold architecture and mechanical properties. Biomaterials can also act as drug depots, providing protection and enabling sustained release of therapeutic cargoes. While countless approaches exist in the literature, we highlight here examples of microrheology-enabled design and characterization of scaffolds for tissue engineering and hydrogel-based drug delivery vehicles.

**FIG. 5.**
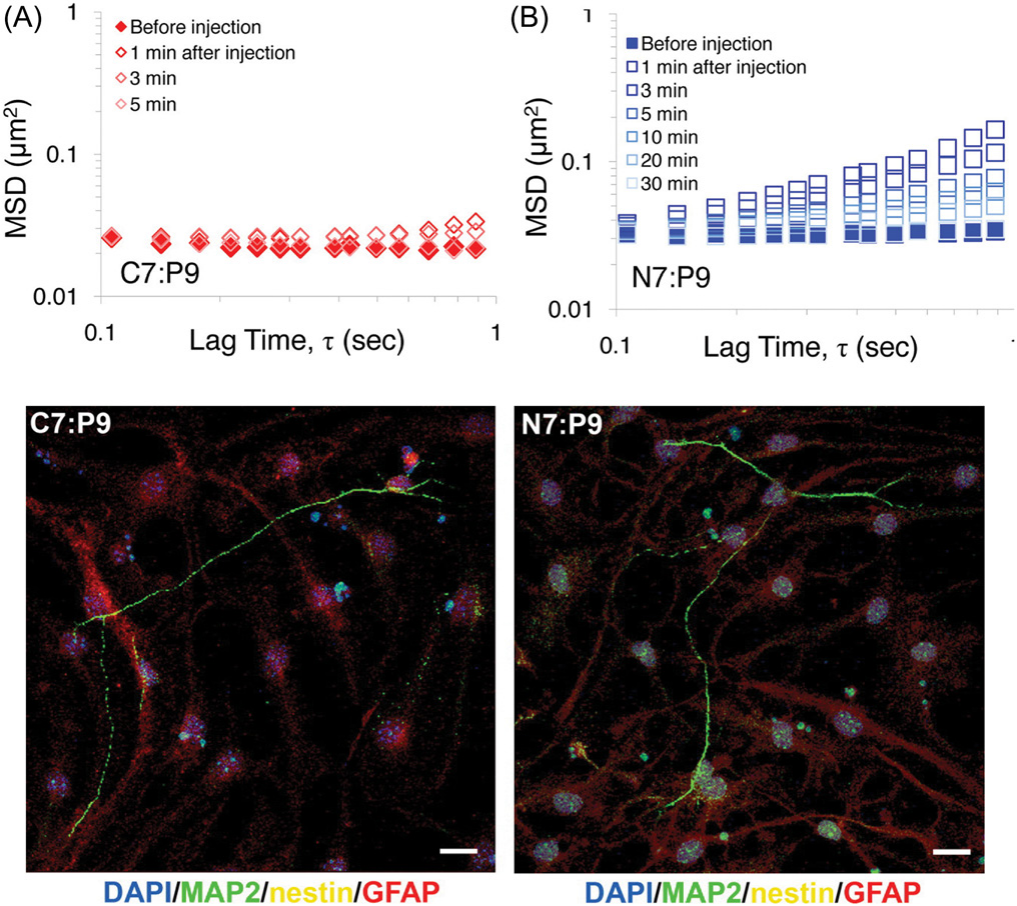
Designing injectable peptide biomaterials for regenerative medicine using microrheology. (a) and (b) Shear-thinning and self-healing properties of two peptide hydrogel formulations after syringe injection determined based on measured MSD. (Lower panel) Each gel supported differentiation of adult neural stem cells. Reproduced with permission from Wong Po Foo *et al.*, Proc. Natl. Acad. Sci. U. S. A. **106**, 22067 (2009). Copyright 2009 National Academy of Sciences of the United States of America.

Novel approaches to design scaffolds for tissue engineering have been realized using microrheology to determine the mode of gelation. For example, Yamaguchi *et al.* designed a novel strategy to cross-link PEG-based scaffolds through heparin binding to vascular endothelial growth factor (VEGF), which leads to releasing when exposed to VEGF receptor 2 (VEGFR-2).^121^ Addition of VEGF to heparin-functionalized PEG formed a viscoelastic network as confirmed by optical tweezers-based microrheology. This strategy leads to enhanced proliferation of VEGFR-2 positive aortic endothelial cells when seeded within these biomaterial scaffolds. A novel injectable peptide-based biomaterial for cell encapsulation was designed to assemble through transient cross-linking domains.^122^ Microrheology was used as a high-throughput tool to determine the peptide concentrations and amino acid sequences that enable their self-assembly into a viscoelastic gel network. While feasible to perform such screening of peptide libraries using bulk rheometers, microrheology provided a rapid and cost-effective means to study peptide biomaterial formation. The shear-thinning and self-healing properties of these peptide scaffolds were also demonstrated by measuring microrheology of the gels after injection through a syringe and monitoring their subsequent recovery over a 30-min period [Figs. 5(a) and 5(b)]. To examine self-healing properties of biomaterials on these short time scales, on the order of minutes, microrheology provided a simple approach to understand the recovery kinetics of peptide biomaterials following injection. Once optimized, these gels supported growth and sprouting of neurites in encapsulated adult neural stem cells (Fig. 5, lower panel).

Given their multipotent capacity, mesenchymal stem cells (MSCs) are often incorporated into these scaffolds where degradation and further remodeling of the matrix occur as they expand and differentiate into specialized cell types. Thus, understanding the degradation process in these scaffolds has important implications into biomaterial design. To explore this, live cell imaging has been used in conjunction with microrheology to locally measure changes in stiffness in degradable PEG-based hydrogels with embedded MSCs that secrete enzymes that degrade the scaffold over time.^105,123^ It was shown that cell motility within these scaffolds is dependent on local degradation to the matrix where the extent of degradation depends on the radial distance from the cell. Previous work has also studied expected microviscosity experienced by cells migrating individually and collectively in pullulan/dextran biomaterial scaffold. To accomplish this, individual cells and cell aggregates were magnetized by loading with magnetic nanoparticles and pulled unidirectionally through the scaffold using magnetic tweezers to determine the apparent viscosity.^124^ When in aggregated form, these cells experienced roughly 10-fold higher viscosities, which may influence their distribution once seeded into tissue constructs. Microrheology also provides a tool to understand how these scaffolds may lead to biophysical changes of encapsulated cells. This has been studied in previous work where MSCs exhibited significant changes in intracellular stiffness as measured by microrheology depending on substrate stiffness.^125^ These intracellular biomechanical changes were shown to play a role in their regenerative capacity and efficacy in a wound closure *in vivo* mouse model.^126^

Biomaterials may also be used for delivery of biologic agents, nanoparticles, and/or small molecule drugs administered orally, topically, or via injection to the site of injury. Using the previously described combined microfluidic microrheology approach, Wu *et al.* showed the reversibility of gel assembly at neutral pH and gel disassembly at acidic pH of hydrazone-functionalized PEG hydrogels to enable drug release when orally administered.^127^ Self-assembly of a novel dual liposome and nanogel biomaterial was verified using microrheology with the ability to enable simultaneous delivery of both nucleic acid and protein-based therapeutics.^128^ Given the complexity of this formulation,^128^ microrheology again provided a cost-effective means to interrogate these nanogel biomaterials and optimize conditions for their successful formulation. Moreover, nano- and microparticles in these types of composite biomaterial formulations may be used as markers if made fluorescent to understand biomaterial deformation in combination with passive or active microrheology.^23,129^ We also note that determination of the heterogeneity in the network size using microrheology will be informative in design of these systems in future work given its importance to drug release. Biological hydrogels can also be engineered to selectively immobilize potentially harmful pathogens such as viruses and bacteria, depending on their size and surface chemistry.^130^ For these applications, microrheology can be used to characterize the gel network size and design biomaterials with the ability to physically or chemically entrap target pathogens. For example, microrheological measurements were performed on pH-responsive hydrogels that enabled capture of HIV at acidic pH within the cervicovaginal tract and potentially offer a means to prevent HIV transmission.^131^ In addition, microrheology was used to develop IgG-loaded biological gels composed of matrigel, laminin, and entactin as barriers to virus-like nanoparticles and bacteria.^132^ It was demonstrated that these barrier hydrogels reinforced with IgG specific to *Salmonella* reduced bacterial penetration to the underlying tissue and significantly reduced infectivity.

## V. SUMMARY AND FUTURE OUTLOOK

Microrheology is widely appreciated in the fields of colloid science and biophysics but has gained less attention in biomaterials research. The information gathered using this approach can be powerful for those creating complex biomaterials with a nonuniform structure that are often difficult to characterize using traditional approaches. Screening of biomaterial designs can be achieved in a rapid and cost-efficient manner using microrheology. Cell-laden biomaterials can also be characterized in real time to study biomaterial degradation and remodeling. Importantly, an abundance of data exists for microrheological characterization of biological systems *in vitro, in vivo*, or *ex vivo*, which can be leveraged as specifications in the design of physiological models.^95,133–137^ Importantly, this technique can be performed using equipment standard to most bioengineering labs and data interpretation is straightforward for nonexperts. In the future, microrheology could expand biomaterials beyond their traditional use as ECM scaffolds and drug delivery vehicles. For instance, the designs of synthetic organelles, extracellular traps, and biofilms are additional applications for biomaterials in which microrheology could potentially be utilized.^138–140^ These and many other biological systems are difficult to fully characterize using traditional rheological methods alone. Hence, measuring properties on the micro- to nanoscale via microrheology can enhance our knowledge and abilities to emulate complex biological systems.

## Data Availability

None of the data included in this review is original. Please contact the respective journals for inquiries on data availability from the previously published figures included in our review.
